# Transradial Perilunate Fracture-Dislocation With Lunotriquetral Coalition

**DOI:** 10.7759/cureus.115625

**Published:** 2026-09-01

**Authors:** Manuel Cerdeiros, Olga Tkachuk Correia, Ana Baía, Diogo Mestre, André Ramos

**Affiliations:** 1 Orthopaedics and Trauma, Unidade Local de Saúde do Baixo Alentejo, Beja, PRT

**Keywords:** carpal coalition, carpal instability, lunotriquetral synostosis, perilunate fracture-dislocation, transradial fracture

## Abstract

Perilunate fracture-dislocations are devastating injuries, and their occurrence in the presence of a congenital carpal coalition is exceedingly rare. This combination fundamentally alters normal wrist biomechanics and significantly complicates surgical decision-making. We present a case of a 52-year-old female with acute hand ischemia following a high-energy motorcycle collision. Radiographic evaluation revealed a transradial perilunate fracture-dislocation complicated by a unilateral Minnaar type 3 congenital lunotriquetral coalition. Emergent closed reduction was performed, which immediately restored distal perfusion. The patient subsequently underwent minimally invasive percutaneous fixation. At the 12-month follow-up, she exhibited persistent radiocarpal pain, a severely restricted arc of motion, and radiographic evidence of carpal subluxation with early post-traumatic arthrosis. The coexistence of a transradial fracture-dislocation and a complete lunotriquetral coalition alters typical trauma kinematics, concentrating massive deforming forces across the intact intervals. While prompt closed reduction is critical for neurovascular salvage, this case highlights that selective percutaneous fixation is inadequate for maintaining long-term stability in such complex variants. Rigid internal fixation and robust capsuloligamentous repair are likely necessary to prevent rapid joint degeneration.

## Introduction

Perilunate fracture-dislocations and their transosseous variants (e.g., transradial and transscaphoid) are complex articular injuries typically resulting from high-energy trauma [1,2]. The pathophysiology is classically described by the Mayfield classification, which delineates the progressive failure of pericarpal ligamentous stabilizers in response to axial loads applied to a hyperextended, ulnarly deviated, and supinated wrist [3]. Stage IV denotes maximum instability, culminating in volar lunate dislocation with global disruption of the intrinsic (scapholunate and lunotriquetral) and extrinsic carpal ligaments [3,4].

Congenital carpal coalition is a rare anatomical anomaly with an estimated prevalence of 0.1%, arising from an embryonic failure of articular mesenchyme cavitation [5]. Lunotriquetral synostosis accounts for nearly 90% of all carpal coalitions and is stratified by the Minnaar classification, wherein type 3 designates complete osseous fusion [6,7].

The occurrence of a transradial perilunate fracture-dislocation in the setting of a Minnaar type 3 coalition is a biomechanical and clinical rarity with sparse documentation in the literature. This case highlights the profound alteration in force dissipation caused by this anatomical anomaly during trauma, as well as the inherent challenges and potential complications in determining the optimal surgical strategy for carpal stabilization.

## Case presentation

A 52-year-old, otherwise healthy female motorcyclist was admitted to the emergency department following a high-energy blunt trauma to her right wrist. She presented approximately two hours post injury. Clinical examination revealed severe structural wrist deformity associated with acute neurovascular compromise: a cyanotic hand, absent capillary refill, unpalpable distal pulses, and generalized paresthesias in the median nerve distribution. Radiographs demonstrated a volar perilunate fracture-dislocation with a transradial fracture line, consistent with a Mayfield stage IV injury [3]. Additionally, a complete lunotriquetral synostosis was identified, a Minnaar type 3 congenital coalition [6], giving the proximal carpal row an elongated, monobloc structure (Figure 1). Contralateral radiographs confirmed the anomaly was unilateral, with the left carpus demonstrating normal physiological segmentation.

**Figure 1:**
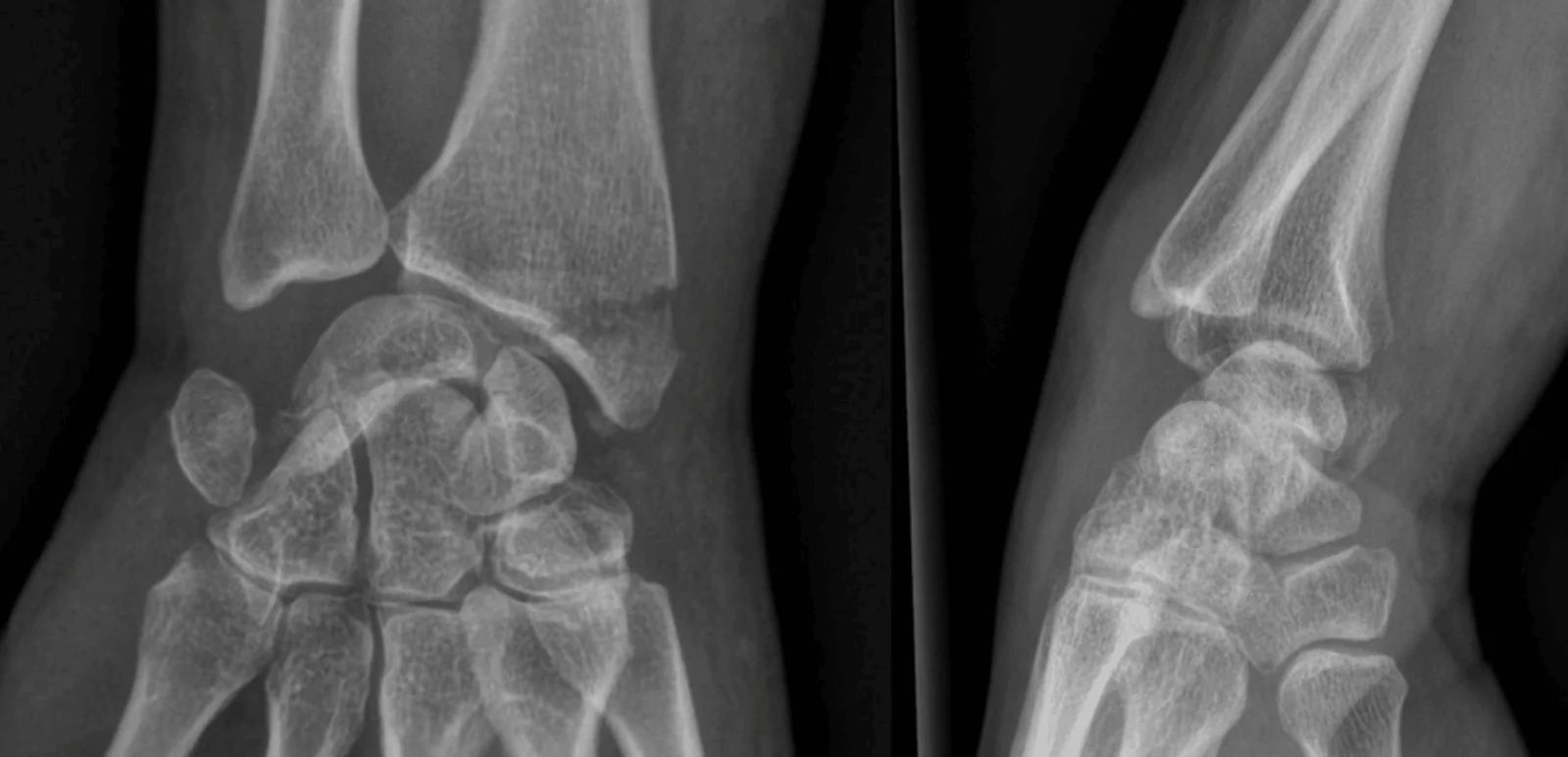
Initial injury radiographs of the right wrist. Anteroposterior (left) and lateral (right) projections demonstrating a transradial perilunate fracture-dislocation. The anteroposterior view reveals a transradial fracture line and a complete congenital osseous fusion between the lunate and triquetrum (Minnaar type 3 coalition), forming a rigid monobloc. The lateral view confirms the severe structural instability with volar dislocation of the carpal block, consistent with a Mayfield stage IV injury.

Given the critical ischemia and impending risk of irreversible necrosis, an emergent closed reduction was performed [8]. This maneuver provisionally restored carpal alignment, resulting in the immediate reversal of ischemia, robust tissue reperfusion, and marked improvement in neurological symptoms (Figure 2).

**Figure 2:**
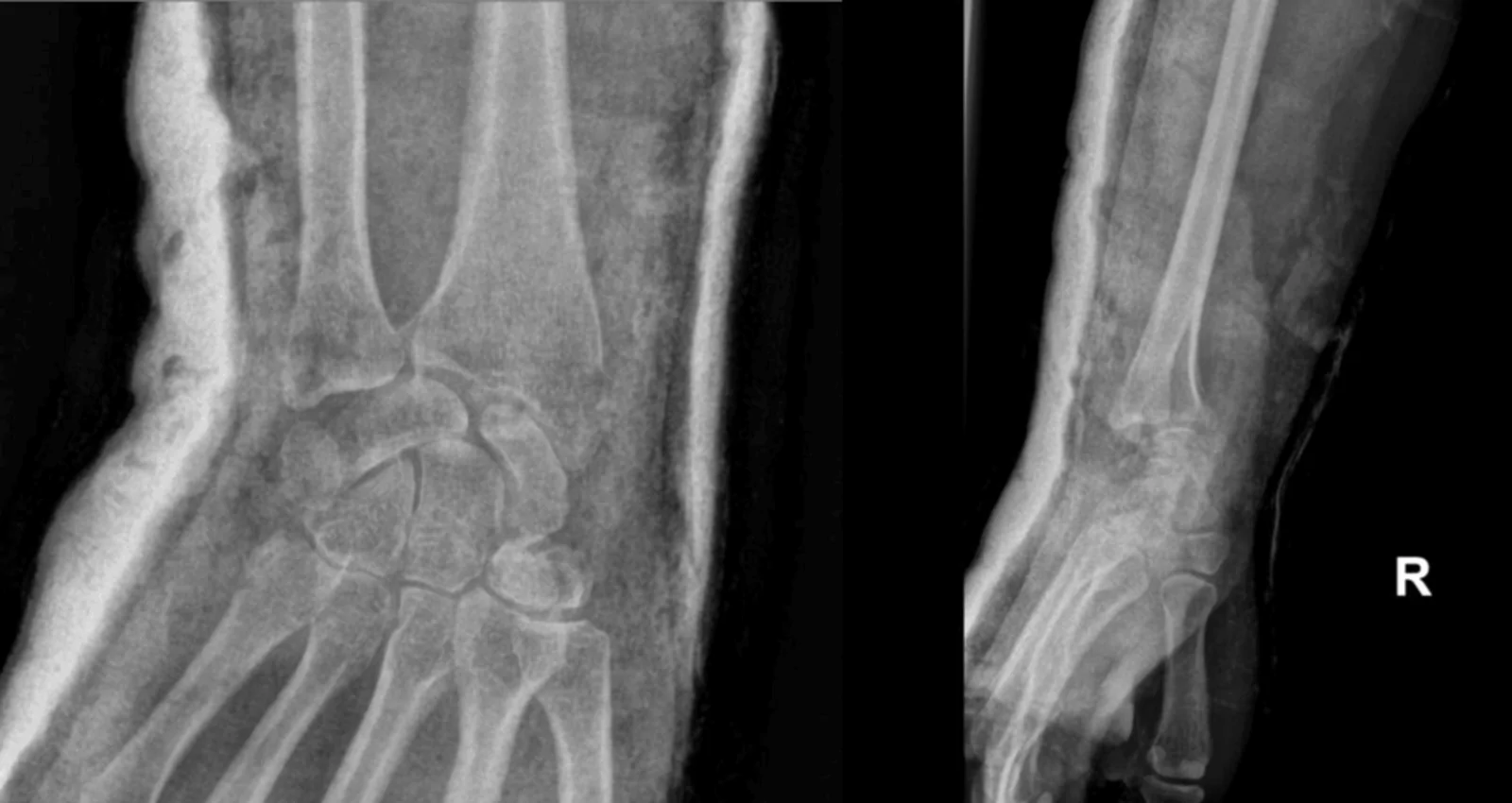
Post-reduction radiographs of the right wrist. Anteroposterior (left) and lateral (right) projections were taken in a temporary splint following emergent closed reduction. The imaging demonstrates provisional restoration of carpal alignment, with the lunotriquetral monobloc relocated over the distal radius. This maneuver successfully relieved the acute neurovascular compromise prior to definitive surgical fixation.

Following stabilization of the vascular emergency, a computed tomography (CT) scan of the wrist was acquired for preoperative planning. CT confirmed the complex transradial fracture and complete lunotriquetral coalition, while definitively ruling out any occult fractures of the scaphoid. However, the imaging revealed significant intercarpal instability, necessitating surgical fixation.

Twelve hours following the initial closed reduction, the patient was taken to the operating room. Under regional anesthesia and fluoroscopic guidance, percutaneous reduction and osteosynthesis were performed. The transradial fracture was stabilized using an autocompressing cannulated screw. The anomalous lunotriquetral complex, functioning as a single osseous unit, was anatomically reduced relative to the scaphoid and stabilized percutaneously with two transverse Kirschner wires (K-wires) (Figure 3). A deliberate decision was made to omit radiolunate transfixation wires to prevent iatrogenic chondral injury [9]. The limb was immobilized in a dorsal short-arm splint.

**Figure 3:**
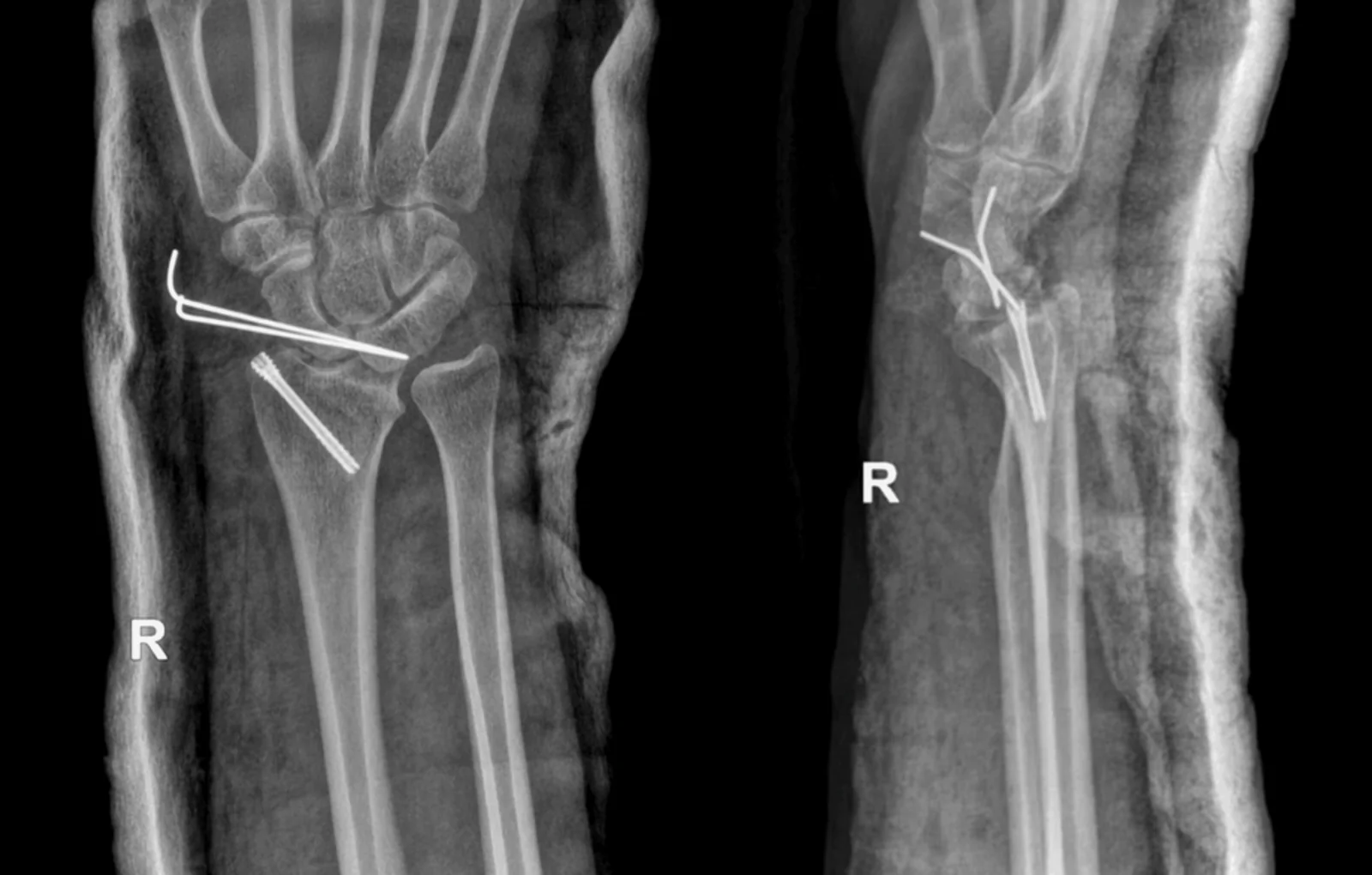
Postoperative radiographs of the right wrist following percutaneous fixation. Anteroposterior (left) and lateral (right) projections demonstrating the minimally invasive fixation construct. The transradial fracture is anatomically reduced and stabilized with a single autocompressing cannulated screw. The scapholunate interval is stabilized with two transverse percutaneous Kirschner wires (K-wires) anchoring the scaphoid to the lunotriquetral monobloc.

At the two-week postoperative evaluation, the patient reported adequate pain control, and radiographs confirmed the maintenance of articular alignment. At six weeks, the K-wires and immobilization were removed in the outpatient clinic, and a functional rehabilitation protocol was initiated.

At the 12-month postoperative follow-up, the patient reported persistent, activity-limiting pain in the radiocarpal joint. Objective physical examination using a goniometer demonstrated a significantly restricted arc of motion, with wrist extension measuring 30° and flexion measuring 25°. Grip strength was reduced to approximately 60% of the uninjured contralateral limb. Patient-reported outcome measures reflected this poor functional recovery, yielding a QuickDASH (Quick Disabilities of the Arm, Shoulder, and Hand) score of 52 [10].

Furthermore, follow-up radiographs revealed a loss of anatomical carpal alignment with volar subluxation of the carpal block, widening of the scapholunate interval, and subchondral sclerosis consistent with early post-traumatic radiocarpal arthrosis (Figure 4).

**Figure 4:**
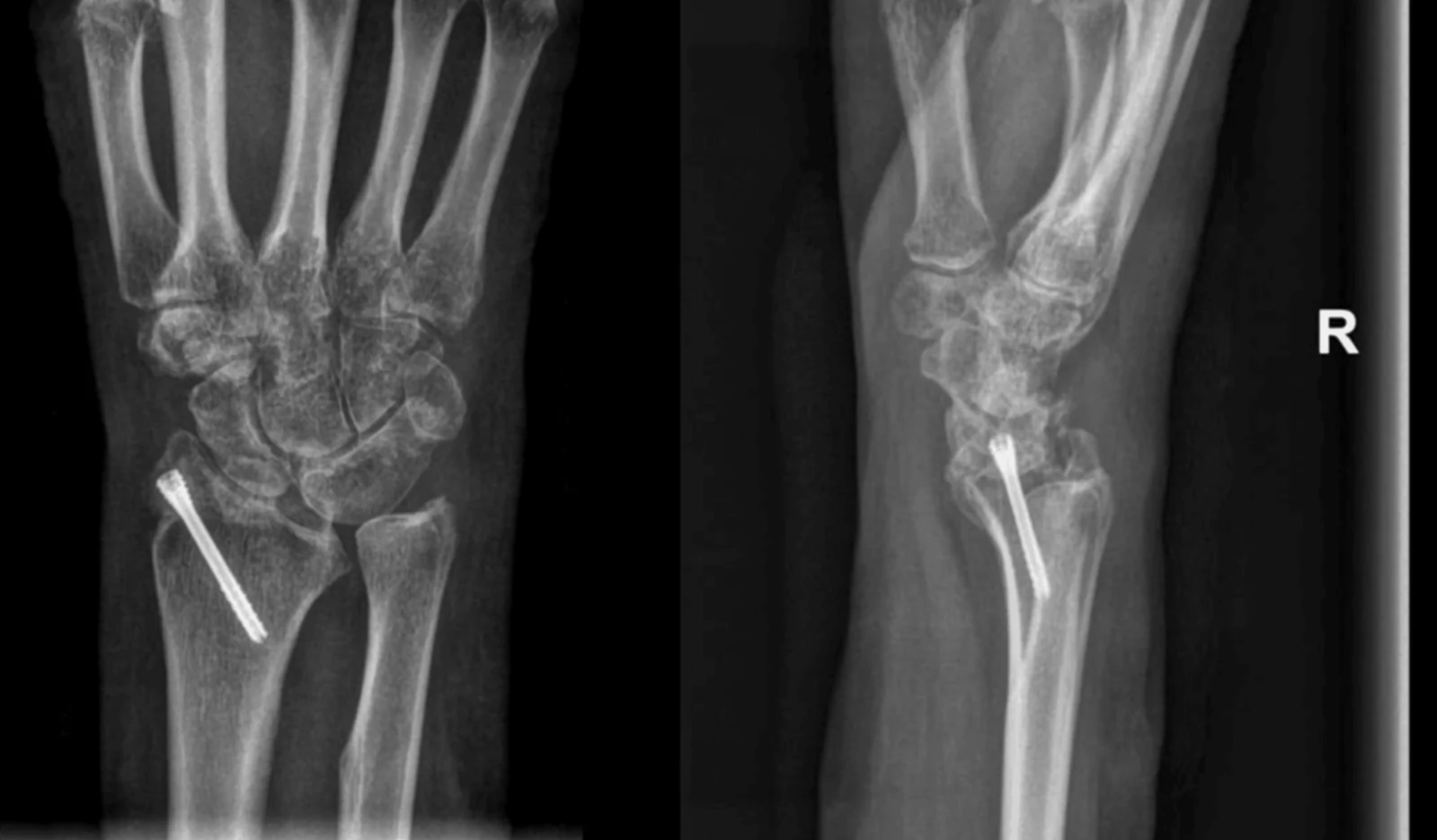
Twelve-month postoperative radiographs of the right wrist. The anteroposterior view (left) demonstrates widening of the scapholunate interval and subchondral sclerosis. The lateral view (right) reveals a loss of anatomical carpal alignment with volar subluxation of the carpal block. Together, these findings are consistent with capsuloligamentous failure and early post-traumatic radiocarpal arthrosis.

## Discussion

The biomechanics of this case represent a highly unusual injury pattern. In a classic perilunate fracture-dislocation, the deforming force propagates sequentially through the scapholunate interval and subsequently through the lunotriquetral space [3]. In this patient, the presence of a Minnaar type 3 congenital coalition fundamentally altered the trauma kinematics: the complete osseous fusion caused the lunate and triquetrum to function as a rigid monobloc [5,6]. Consequently, the force was entirely directed into the radial column and the scapholunate ligament complex, resulting in transradial fracture and en bloc volar dislocation.

The timing of intervention was of paramount prognostic importance. Emergency reduction of the dislocation within a two-hour window successfully interrupted the pathophysiological cascade, reversing the acute ischemia and mitigating the risk of irreversible neuropathy [2,8].

However, the definitive management of this injury proved inadequate for long-term functional preservation. Current literature predominantly advocates for open reduction and internal fixation (ORIF) in the management of perilunate dislocations, emphasizing direct repair of intrinsic ligaments and the articular capsule to restore carpal stability [2]. In an attempt to minimize soft-tissue trauma and preserve the radiocarpal articular surface, a minimally invasive percutaneous approach was initially selected [9].

The fixation strategy was based on the rationale that a Minnaar type 3 synostosis provides absolute intrinsic stability across the lunotriquetral joint, leaving the K-wires dedicated solely to stabilizing the scapholunate interval. Furthermore, articular transfixation with a radiolunate K-wire was deliberately omitted to avoid the well-documented risk of chondral iatrogenesis [9,11].

While complex overlapping shadows on one-year follow-up radiographs might suggest unrecognized bony pathology to an outside observer, the perioperative CT scan definitively confirmed an intact scaphoid. Therefore, the rapid development of radiocarpal arthrosis and subluxation at 12 months is attributed purely to profound capsuloligamentous incompetence. The poor clinical outcome, highlighted by severe stiffness, chronic pain, and early joint failure, suggests that our surgical rationale underestimated the massive instability inherent to this specific injury pattern.

## Conclusions

By avoiding open ligamentous repair and radiolunate transfixation, we failed to provide the necessary mechanical support to counteract the deforming forces during the healing phase. This case demonstrates that while closed reduction percutaneous pinning may be tempting to preserve biology and avoid iatrogenic cartilage damage, highly unstable perilunate fracture-dislocations complicated by altered carpal kinematics likely require the rigid stability afforded by ORIF and robust capsuloligamentous repair.
